# Association between nuclear factor of kappa B (NFκB) deficiency and induction of eryptosis in mouse erythrocytes

**DOI:** 10.1007/s10495-020-01644-y

**Published:** 2020-11-16

**Authors:** Mehrdad Ghashghaeinia, Ulrich Mrowietz, Peter Dreischer, Martin Köberle

**Affiliations:** 1 Department of Dermatology, Psoriasis-Center, University Medical Center Schleswig-Holstein, Campus Kiel, Rosalind-Franklin-Str. 7, 24105 Kiel, Germany; 2 Physiologisches Institut, Abteilung für Vegetative und Klinische Physiologie, Eberhard Karls University of Tübingen, 72074 Tübingen, Germany; 3 Department of Dermatology and Allergology, School of Medicine, Technical University of Munich, Biedersteinerstr. 29, 80802 München, Germany

**To the Editors,**

Hemoglobin makes up 95% of the total proteins in human erythrocytes. This fact was fatal for the erythrocytes and they were degraded as cells responsible only for respiratory gas exchange (CO_2_ and O_2_). However, in concert with white blood cells (WBCs), human erythrocytes play a vital role in immune and defence mechanisms. Complement 3b and complement receptor 1 (CR1) mediate attachment of erythrocytes to bacteria, lead to phagocytosis of erythrocyte-bound bacteria in liver and spleen and the return of intact erythrocytes into the circulation. Although the CR1 occurrence on a single neutrophil is four times higher than on a single erythrocyte, the sheer number of erythrocytes is the decisive factor in removing the vast majority of bacteria from the circulation by means of the erythrocyte immune system [1]. Following microbial contact, the bronchial epithelial cells express the inflammatory cytokine IL-36, thereby strengthening host defense. Human dendritic cells (DCs) constitutively express IL-36 receptor. IL-36 cytokines (IL-36α, -β and -γ) initiated maturation of DCs and the resulting NFκB-dependent IL-12 production induces T_H_1-polarization of naive CD4^+^ T cells and IFN-γ secretion. Interestingly, heme binding to IL-36α impairs IL-36-mediated signaling pathways [2] and human erythrocytes inhibit DCs maturation while circulating in peripheral blood. Therefore, the transport of bacteria by erythrocytes and the delivery of these pathogens to macrophages residing in spleen and liver in combination with the sequestration of IL-36 is an effective way to relieve the host’s immune system and at the same time to avoid the excessive inflammatory response. Induction of expression of the chemokine IL-8 and NFκB activation by IL-36γ, NFκB-mediated IL-8 expression and its association with tumor angiogenesis, the existence of a unique chemokine receptor on human erythrocyte capable of binding an array of chemokines including IL-8, rapid and vast binding of IL-8 by erythrocytes to avoid IL-8 mediated neutrophils stimulation and inhibition of monocytes-drived IL-8 production by human erythrocytes show the complexity of erythrocytes biology. Both mouse and human erythrocytes function as a sink for sphingosine-1-phosphate (S1P) and release on demand this molecule into the blood plasma through a finely regulated mechanism; S1P impairs lymphocytes circulation. These data show that human erythrocytes have not only respiratory but also anti-inflammatory, immuno-modulatory and anti-tumoral functions. Human erythrocytes possess the transcription factor NFκB and it exists a direct correlation between NFκB abundance and erythrocytes’ survival [3]. The evolutionarily conserved transcription factor family of NFκBs: p105/50 (NFκB1), p100/p52 (NFκB2), p65 (Rel-A), Rel‐B and c‐Rel composed of homo and heterodimers proteins regulating the gene expression of over 200 proteins involved in apoptosis, cell growth, inflammation, cell-to-cell interactions, angiogenesis and immune response. To further investigate the aforementioned correlation (i.e. the linkage between NFκB and survival in anucleated erythrocytes) we used 8-weeks old female NFκB-p50 knock-out and congenic wild-type C57BL/6 mice. 200 µl of blood were retrobulbarly collected from each of 20 female mice and placed in EDTA tubes; ten NFκB-p50 -deficient and ten congenic C57BL/6 wild-type mice. In the next step, blood samples were washed twice in PBS solution (without Ca^2+^ and Mg^2+^) and after each round the supernatant and the upper layer of RBCs were discarded. Finally, 5 µl of the corresponding RBCs pellets were resuspended in 5 ml Ringer solution (0.1% Hct) and incubated at 37 °C for the following time points: 0, 24 and 48 hours. For the indicated time points, triplicates each containing 220 µl RBC suspension (~ 2.2 × 10^6^ cells) were taken from the respective samples, transferred to tubes containing 500 µl annexin wash buffer, vortexed and centrifuged. Following removal of the respective supernatants, each cell pellet was vortexed gently but thoroughly to get single cell suspension, stained with annexin V-FITC antibody and eryptosis was measured on the FACS Calibur in FL-1 channel. For each single sample a total of 50,000 cells were counted. Detailed information about the composition of the Ringer’s solution, annexin wash buffer and the staining procedure have already been described by Ghashghaeinia et al. [4]. Indeed, we observed a direct correlation between NFκB-deficiency and increased eryptosis, i.e. programmed cell death of erythrocytes (Fig. 1a and b). It is to note that the original mean value of the number of WBCs in the knockout mice group was three times higher than in the wild type mice group, while the number of erythrocytes (RBCs) was the same in both groups. The total ratio of RBCs to WBCs from 1916:1 for wild type mice group to 650:1 for knock-out mice (Fig. 1c). This could indicate systemic inflammation in NFκB-p50 deficient mice as does the splenomegaly we observed (Fig. 1d). It is known that NFκB-p50 homodimers are refractory to inflammation while NFκB heterodimers exert an inflammatory function. Figure 1e compares spleen and body weight in both NFκB-p50 knock-out mice and control group. NFκB is a positive physiological regulator of glycolysis and glutathione (GSH) synthesis machinery. Obesity associated chronic low-grade inflammation and NFκB activation, go hand in hand. GSH depletion inhibits diet-induced obesity and impairs DNA synthesis. These findings indicate that the application of NFκB inhibitors, GSH depletors or impairment of glucose-6-phosphate dehydrogenase-dependent GSH regeneration could be a potential alternative to treat obesity [5]. The following review illustrates the relationship between the anti-inflammatory effects of insulin and the pro-inflammatory effects of glucose with NFκB as a common target [6]. Our present study underscores the anti-eryptotic function of NFκB-p50 subunit in mice erythrocytes and provides a connection between genomic and non-genomic research. Further in-depth studies will shed light on the underlying mechanisms.

**Fig. 1:**
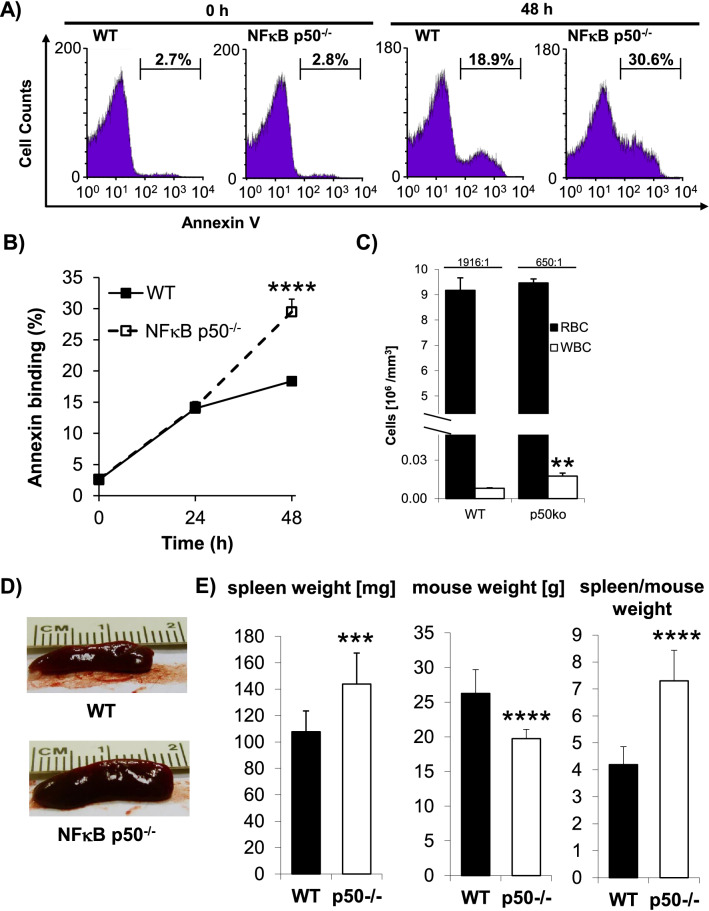
Induction of eryptosis in NFκB-p50 deficient mice is associated with splenomegaly and reduction of body weight. **a** Original histograms of annexin-V binding. **b** Eryptosis curve of NFκB-p50 knock-out mice compared with control group. **c** Increased white blood cells (WBCs) count in transgenic mice compared to control group (**P < 0.01). **d** Photographs showing splenomegally in transgenic mice. **e** Reduction of both spleen and body weight in NFκB-p50 knock-out mice compared with the control group. Number of mice in each group: 10

**Statistical analysis**

Data are presented as the mean values ± SEM of 20 mice. One-way ANOVA with Dunnet’s post test (Fig. 1b) and two-tailed *t* tests were used for statistical analysis. Differences of the means were considered to be statistically significant when the calculated p value was less than 0.05 (**P < 0.01; ***P < 0.001; ****P < 0.0001).

## References

[1] Craig ML, Bankovich AJ, Taylor RP (2002). Visualization of the transfer reaction: tracking immune complexes from erythrocyte complement receptor 1 to macrophages. Clin Immunol.

[2] Wissbrock A, Goradia NB, Kumar A (2019). Structural insights into heme binding to IL-36alpha proinflammatory cytokine. Sci Rep.

[3] Ghashghaeinia M, Cluitmans JC, Toulany M (2013). Age sensitivity of NFkappaB abundance and programmed cell death in erythrocytes induced by NFkappaB inhibitors. Cell Physiol Biochem.

[4] Ghashghaeinia M, Koralkova P, Giustarini D, Mojzikova R, Fehrenbacher B, Dreischer P, Schaller M, Mrowietz U, Martínez-Ruiz A, Wieder T, Divoky V, Rossi R, Lang F, Köberle M (2020). The specific PKC-α inhibitor chelerythrine blunts
costunolide-induced eryptosis. Apoptosis.

[5] Ghashghaeinia M (2019) Pharmaceutical composition containing Bay 11-7082, parthenolide or a combination thereof for the treatment of obesity or cardiovascular diseases. US Patent 10,420,746, B2

[6] Dandona P, Chaudhuri A, Ghanim H, Mohanty P (2006). Anti-inflammatory effects of insulin and the pro-inflammatory effects of glucose. Semin Thorac Cardiovasc Surg.

